# Supplementary material for: Content-rich biological network constructed by mining PubMed abstracts
Source: BMC Bioinformatics. 2004 Oct 8;5:147. doi: 10.1186/1471-2105-5-147 (PMC528731; doi:10.1186/1471-2105-5-147)
Supplement: Additional File 2 — The original results of the above study (non-essential files are deleted to keep the file size under the limit set by BMC bioinformatics). [file 1471-2105-5-147-S2.bz2 › chilibotAdditionalFile2/dip05/457ID10733106E10474/html/CCR3_CCL7.html]

 


 **CCR3** and **CCL7** 
  
Found 58 abstracts in PubMed, retrieved 05.  
 

 What does Google say? 
 PDF only 
| .edu only 

---

**Interactive relationship** (e.g. stimulation, inhibition, etc)

**Non-interactive relationship** (e.g. studied together, co-existance, homology, etc.)

- Our findings point to a role for C C chemokine receptor 2 CCR2 and  **CCR3**  agonists such as monocyte chemotactic proteins MCPs 1 CCL  **CCL7**  and 5 CCL12 as important participants that are subject to regulation by Th2 cytokines interleukin IL 4 and IL 13.  Ref: 12102713 Parasite Immunol, 2002
- We investigated tissue samples and tumor cell suspensions of patients with CD30 CTCL and CD30 CTCL for expression of the chemokine receptors  **CCR3** , CCR4, and CCR8 and the  **CCR3**  ligands eotaxin CCL11, monocyte chemoattractant protein 3  [ **CCL7** ]   **CCL7** , and RANTES CCL5.  Ref: 12393570 Blood, 2003
